# Leptin haploinsufficiency exerts sex-dependent partial protection in SOD1^G93A^ mice by reducing inflammatory pathways in the adipose tissue

**DOI:** 10.1038/s41598-024-52439-z

**Published:** 2024-02-01

**Authors:** Luis C. Fernández-Beltrán, Zeinab Ali, Angélica Larrad-Sanz, Juan I. Lopez-Carbonero, Juan M. Godoy-Corchuelo, Irene Jimenez-Coca, Irene Garcia-Toledo, Liz Bentley, Ulises Gomez-Pinedo, Jordi A. Matias-Guiu, Maria Jose Gil-Moreno, Jorge Matias-Guiu, Silvia Corrochano

**Affiliations:** 1 Neurological Disorders Group, Department of Neurology, Hospital Clínico San Carlos, Instituto de Investigación Sanitaria San Carlos (IdISSC), Madrid, Spain; 2 Department of Medicine, Universidad Complutense de Madrid, Madrid, Spain; 3 Mary Lyon Centre at MRC Harwell, Oxfordshire, UK; 4 Department of Endocrinology and Nutrition, Hospital Clínico San Carlos, Instituto de Investigación Sanitaria San Carlos (IdISSC), Madrid, Spain

**Keywords:** Neurodegenerative diseases, Spinal cord diseases

## Abstract

Amyotrophic lateral sclerosis (ALS) is a fatal neurodegenerative disorder characterized by significant metabolic disruptions, including weight loss and hypermetabolism in both patients and animal models. Leptin, an adipose-derived hormone, displays altered levels in ALS. Genetically reducing leptin levels (Lepob/+) to maintain body weight improved motor performance and extended survival in female SOD1G93A mice, although the exact molecular mechanisms behind these effects remain elusive. Here, we corroborated the sexual dimorphism in circulating leptin levels in ALS patients and in SOD1G93A mice. We reproduced a previous strategy to generate a genetically deficient leptin SOD1G93A mice (SOD1G93ALepob/+) and studied the transcriptomic profile in the subcutaneous adipose tissue and the spinal cord. We found that leptin deficiency reduced the inflammation pathways activated by the SOD1G93A mutation in the adipose tissue, but not in the spinal cord. These findings emphasize the importance of considering sex-specific approaches in metabolic therapies and highlight the role of leptin in the systemic modulation of ALS by regulating immune responses outside the central nervous system.

## Introduction

Amyotrophic lateral sclerosis (ALS) is a fatal motor neuron disease that is characterized by the progressive loss of the upper and lower motor neurons (MNs) at the spinal or bulbar level^1^, leading to muscle atrophy and weakness^2^. ALS usually has a rapid fatal prognosis with no cure, and current pharmacological treatments have limited efficacy^3^. In particular, for patients carrying SOD1 mutations, the current treatment with the antisense oligonucleotide Tofersen is currently being tested in phase 3 clinical trial (NCT04856982). Since ALS is a multifactorial disorder, it is necessary to understand and tackle other disease modifiers and metabolic parameters that influence the clinical course of ALS, so we could apply combinational therapies that might improve the treatment of the patients.

ALS patients typically have a normal or low body mass index (BMI), and lose weight as the disease progresses, which in turn negatively affects the disease prognosis^4^. A low BMI is a risk factor for ALS^5^ and a decrease in BMI after the onset of motor symptoms significantly reduces survival^6^. On the other hand, a higher BMI at diagnosis is associated with a slower disease progression and delayed mortality^7,8^. As a consequence, there are several strategies focusing on increasing the weight of the patients, with some beneficial effects on the fast progressing patients^9^, although longer times and combinations are being conducted to increase those benefits. The causes of weight loss in ALS patients are multifactorial, including reduced food intake due to problems for swallowing (dysphagia), loss of muscle mass (muscle atrophy) and hypermetabolism^10^. Hypermetabolism denotes increased resting energy expenditure (REE) and the origin of hypermetabolism in ALS patients is not clear. Hypermetabolism is observed in half of ALS patients and it is associated with a worse prognosis, faster functional decline and reduced survival^11^. The REE is partly dependent on the body composition and can be centrally regulated in the hypothalamus. Altered hypothalamus regulatory pathways have been described in ALS patients^12^. The hypothalamus regulates body energy balance, food consumption and satiety with complex pathways and hormones such as leptin.

Leptin is an adipocyte-derived hormone that is mainly secreted by the white adipose tissue (WAT), and levels are positively correlated with the amount of body fat^13^. Leptin is an anorexigenic hormone, affecting the hypothalamic pathways that reduce the food intake and increase the energy expenditure. Circulating leptin level serves as a gauge for energy reserves and directs the central nervous system to adjust food intake and energy expenditure. It mediates its actions by binding to leptin receptors (LepRs), which are expressed in the brain and peripheral tissues^14^. Mice that are null for the leptin gene (*Lep*^*ob/ob*^) and humans with congenital leptin deficiency are hypometabolic, hyperphagic, morbidly obese and insulin resistant^15^. Leptin administration in these mice and patients improves hyperglycemia and hyperinsulinemia^16^. Numerous epidemiological studies have implicated leptin in ALS; however, our understanding of the underlying biological mechanisms of the leptin role in the pathogenesis of ALS is limited^17–19^.

Given that leptin is produced mainly in the adipose tissue and, in many advanced ALS cases, the adipose tissue progressively decreases in ALS patients, it was expected that leptin levels in ALS patients would be proportionally lower. Surprisingly, there are variable and conflicting results in clinical leptin measurements. One study reported an increase in leptin levels in ALS patients^20^, two studies showed no significant changes^19,21^ and another two revealed a decrease^18,22^. It has been only recently that some studies (only two out of these five) stratified the levels of circulating leptin by sex, evidencing sexual dimorphism. One of these two recent studies showed that women with ALS had significantly higher leptin levels than controls and men with ALS^21^. The other recent study previously reported a general decrease in circulating leptin levels in ALS, but after stratifying by sex, the decrease was only significant in men with ALS^22^. Thus, the lack of patient stratification (by sex, disease stage or genetics) in most studies and clinical trials may explain the great variability in the different measurements and results in leptin concentration in ALS patients. Leptin levels have also been studied in different animal models of ALS. In the well characterized SOD1^G93A^ ALS mouse model, decreased adipose tissue associated with lower circulating leptin levels were found^23^. But again as in the patients, once the data was stratified by sex, another study reported that this decrease was only significant in male mice^24^.

Those observations have led to the use of leptin in pre-clinical studies as potential disease modifier, both increasing and reducing the levels of leptin. Leptin administration in male TDP-43 A315T mice caused a longer disease duration (due to earlier disease onset) and mild improvements in motor performance^25^, although no extension of survival was observed. In contrast, reducing the levels of leptin in the female SOD1^G93A^ mice, had an effect in the whole-body metabolism (by increasing body weight and fat mass), which was accompanied by improvements in motor symptoms, motor neuron counts and extended survival^24^. While we know that reducing leptin levels in females can maintain the body weight, which is beneficial for the disease prognosis, its exact molecular mechanisms and roles outside the hypothalamus remain unknown.

Here we aimed first to disentangle the association of the levels of circulating leptin in men and women ALS patients and presymptomatic SOD1^G93A^ mice. Second, we aimed to determine the mechanism by which lowering leptin systemically has beneficial effects on SOD1^G93A^ female mice. We generated genetically deficient leptin SOD1^G93A^ mice and studied the transcriptome in the subcutaneous fat depots and in the spinal cord, in order to identify the molecular pathways that might be acting upon the beneficial effects in the disease of the female mice.

## Results

### Sexual dimorphism in serum leptin levels in ALS patients and SOD1^G93A^ mouse model

**Figure 1:**
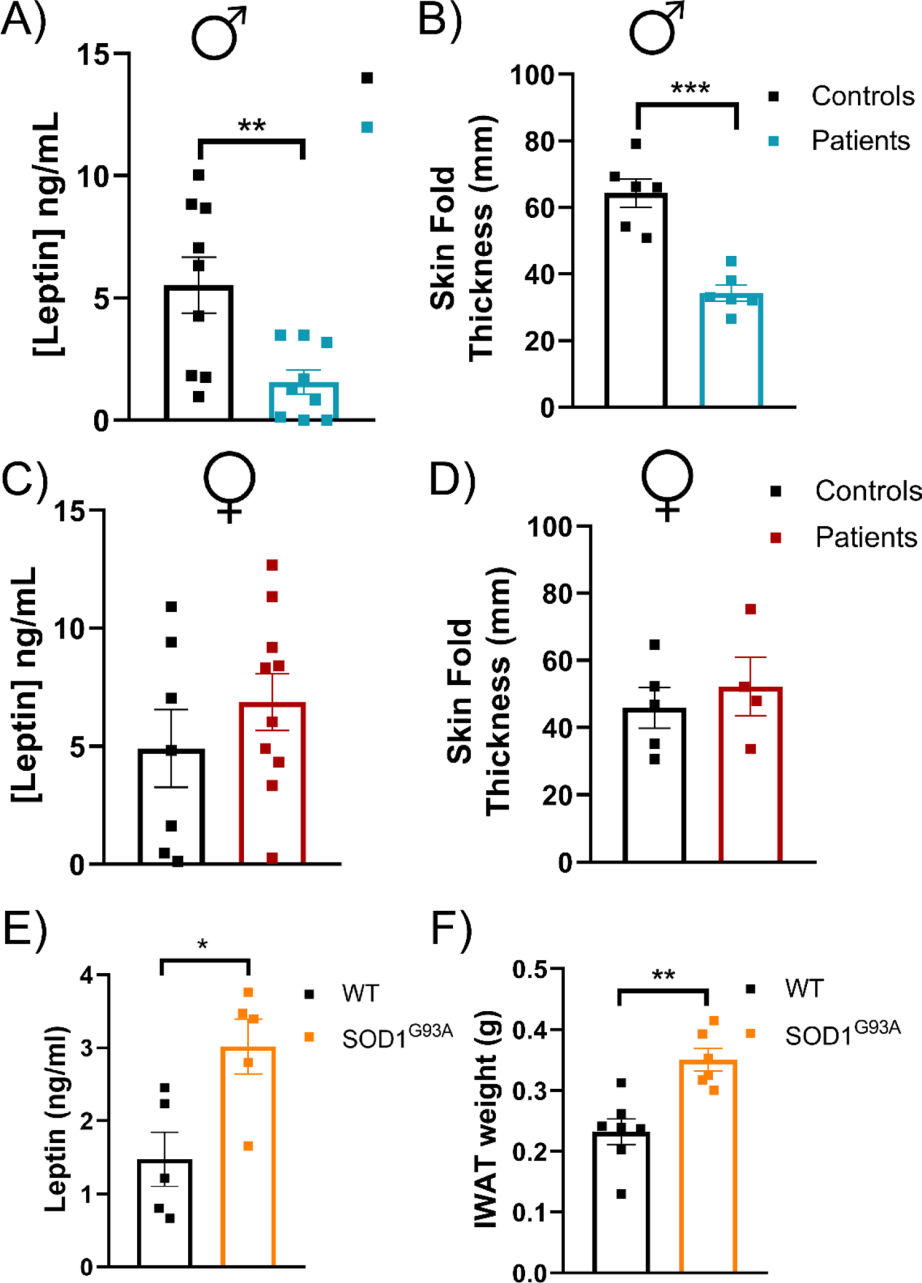
Circulating levels of leptin and subcutaneous fat in ALS and SOD1^G93A^ female mice. Serum leptin concentrations from men (**A**) and women (**C**) controls and ALS patients. Men: controls (n = 9), patients (n = 9). Women: controls (n = 7), patients (n = 10). (**B**, **D**) Indirect estimation of subcutaneous fat using the skinfold thickness (mm) in healthy controls and ALS patients. (**E**) Plasma leptin concentrations from female p90 mice. Females: WT (n = 5), SOD1^G93A^ (n = 5). (**F**) Dissected iWAT weights from female mice at P90 (n = 5). Data is shown as the mean ± SEM and analyzed using the unpaired t-test. *p < 0.05. **p < 0.01.

Circulating levels of leptin in ALS patients have been somehow controversial, but there are studies that point out that the huge variation found might be partly due to the sex differences in ALS patients^22^. In order to elucidate whether leptin levels in ALS are associated with sex, we analyzed the serum of ALS patients and age-sex-matching healthy controls. As previously reported^22^, the levels of leptin differ between men and women in ALS (Fig. 1A) with lower levels of leptin in the blood of men ALS patients and no significant alterations in the women suffering ALS (Fig. 1C). Thus, it supported the idea that the treatment strategy of lowering the levels of leptin in ALS could work in the women but not in the men. Coincidently, previous studies measuring circulating levels of leptin found sexual dimorphism in SOD1^G93A^ mice, with normal levels of leptin in females and much lower levels in males compared to wild-type littermates, at early (90 days, P90) and late (120 days, P120) disease stages^24^. To corroborate this finding, we measured circulating leptin levels by enzyme-linked immunosorbent assay (ELISA) in the blood of females at P90. Female SOD1^G93A^ mice had double the levels of leptin than their wild-type (Fig. 1E).

Since leptin is mainly produced in the subcutaneous white adipose tissue, the circulating levels are normally proportional to the amount of subcutaneous fat depots. We checked whether the levels of leptin in blood were also correlated with these fat depots in ALS patients, and in SOD1^G93A^ mice, despite weighing less than their controls. We used the skinfold thickness measurement as a noninvasive method of body fat estimation in ALS patients. Men suffering from ALS had less amount of subcutaneous fat tissue and this reduction was not similarly observed in women with ALS. As expected, leptin levels correlated with changes in subcutaneous fat tissue (Fig. 1B,D). Inguinal white adipose depots (iWAT) from SOD1^G93A^ and WT littermate female mice at 90 days of age were dissected and weighed (Fig. 1F). Despite of having more body weight, the iWAT depot of SOD1^G93A^ female mice weighed more than those of their control WT littermates, which correlated with the higher levels of leptin in the blood, even when corrected by body weight (data not shown). These results showed that the reduction in body weight, at least in the first stages of disease in the female SOD1^G93A^ mice, was not totally due to a major loss of adipose tissue, which explained the higher levels of circulating leptin in females SOD1^G93A^ mice.

### Leptin haploinsufficiency restores normal levels of circulating leptin and maintains the weight in SOD1^G93A^ females mice

A genetic strategy to lower leptin levels has been previously used in SOD1^G93A^ mice, aiming at ameliorating the disease burden and progression by reducing the systemic hypermetabolism and preserving the body weight^24^. They found that lowering the systemic levels of leptin had beneficial effects in females but not in males SOD1^G93A^. Thus, we aimed to identify the pathways that might be operating to exert beneficial effects on the disease of SOD1^G93A^–*Lep*^*ob*/+^ females, as they could be interesting therapeutic targets. We selected two tissues in which studying those potential pathways: (i) the lumbar spinal cord, to evaluate the direct effect of the leptin haploinsufficiency on the main primary affected tissue in the SOD1^G93A^ mice, and (ii) in the iWAT, as the main producer and receptor tissue of leptin in the body responding to systemic metabolism.

**Figure 2:**
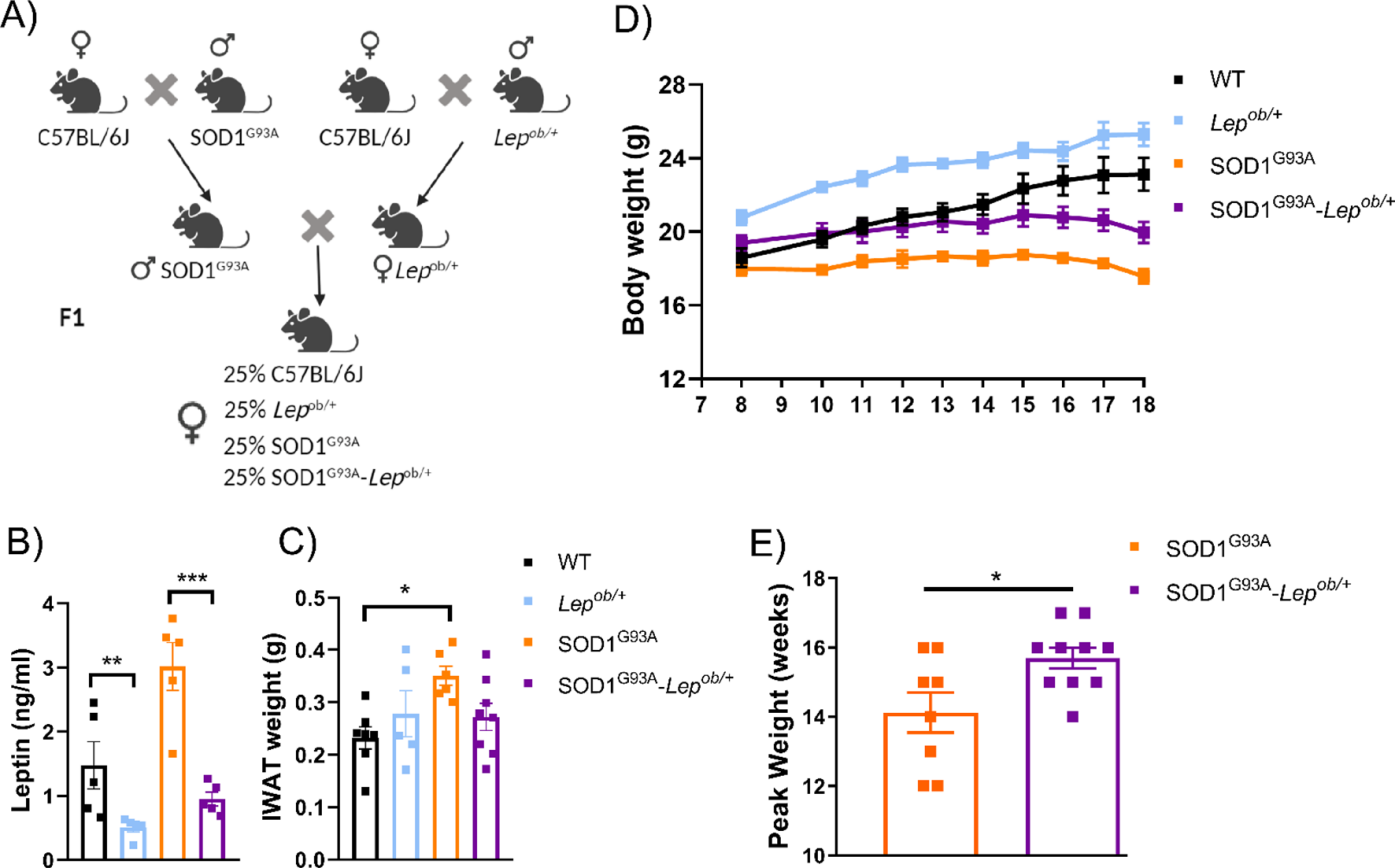
Breeding strategy to generate leptin-deficient mutant SOD1^G93A^ female mice. (**A**) SOD1^G93A^ mice with leptin haploinsufficiency (SOD1^G93A^–*Lep*^*ob/*+^) were generated by crossing female *Lep*^*ob/*+^ with males SOD1^G93A^. Ratios of females born from the F1 intercrossed breeding. The four genotypes are produced in normal ratios. (**B**) Plasma leptin concentrations from female P90 mice (n = 5). (**C**) Weight of the dissected subcutaneous iWAT depots in female P90 mice (n = 7 WT, n = 5 *Lep*^*ob/*+^, n = 6 SOD1 ^G93A^ and n = 8 SOD1^G93A^–*Lep*^*ob/*+^). (**D**) Weekly body weight measurements from 8-weeks old onwards in female mice (**E**) Onset of pathology defined as “age at peak of body weight”, in weeks, in female mice (n = 8 SOD1 ^G93A^, and n = 10 SOD1^G93A^–*Lep*^*ob/*+^). Data is shown as the mean ± SEM and analyzed using the unpaired *t-*test.

We replicated the previously published strategy^24^ and generated SOD1^G93A^ female mice with leptin haploinsufficiency (SOD1^G93A^–*Lep*^*ob/*+^), by crossing female *Lep*^*ob/*+^ with males SOD1^G93A^ (Fig. 2A). We first validated the effectiveness of the leptin-haploinsufficiency background in our mice by measuring serum leptin levels in the four different groups of the study, all females at 90 days of age (P90), before the onset of weight loss. As expected, the amount of leptin in blood was given by their genetics, with reduced levels in the *Lep*^*ob/*+^ mice compared to their *Lep*^+*/*+^ littermates (Fig. 2B). We next weighed the dissected subcutaneous iWAT depots in these mice and found that the iWAT depots were not very different between the groups (Fig. 2C), although there were some reductions in the double mutant compared to the SOD1^G93A^ mice. These results suggest that reducing the circulating levels of leptin had the expected effect in wild-type mice, increasing the fat depots, but the effect seems less evident in the fat depots of SOD1^G93A^–*Lep*^*ob/*+^ mice. Next, we measured the effect of leptin deficiency in the body weight of the mice along the disease progression. The deficiency of leptin seems to maintain the weight of the SOD1^G93A^ female mice, similar to those of the wild-type littermates, especially at early stages of the progression of the disease (Fig. 2D). In addition, one of the possible criteria to define the onset of disease in this mouse model is the time at which peak body weight is reached. Using this parameter, we observed a significant delay at the onset in SOD1^G93A^–*Lep*^*ob/*+^ female mice (Fig 2E). These results evidenced that the iWAT depot weights in the SOD1^G93A^ female mice were not proportional to their body weights, supporting previously reported alterations in the fat tissues of ALS patients, and how the observed weight loss at early disease stages are not necessarily explained by a reduction in the fat tissue in the body^4^.

### Leptin deficiency had a major impact in the iWAT transcriptome in SOD1^G93A^ mice

The iWAT regulation looked altered in SOD1^G93A^ female mice, and the leptin deficiency seemed to be able to preserve or maintain their body and iWAT weights. Thus, we aimed to identify the genes and pathways that are operating in the iWAT and spinal cord of the SOD1^G93A^ in comparison to the SOD1^G93A^–*Lep*^*ob/*+^ mice, in order to understand both the pathological mechanism and how those might change by the leptin deficiency on each tissue.

**Figure 3:**
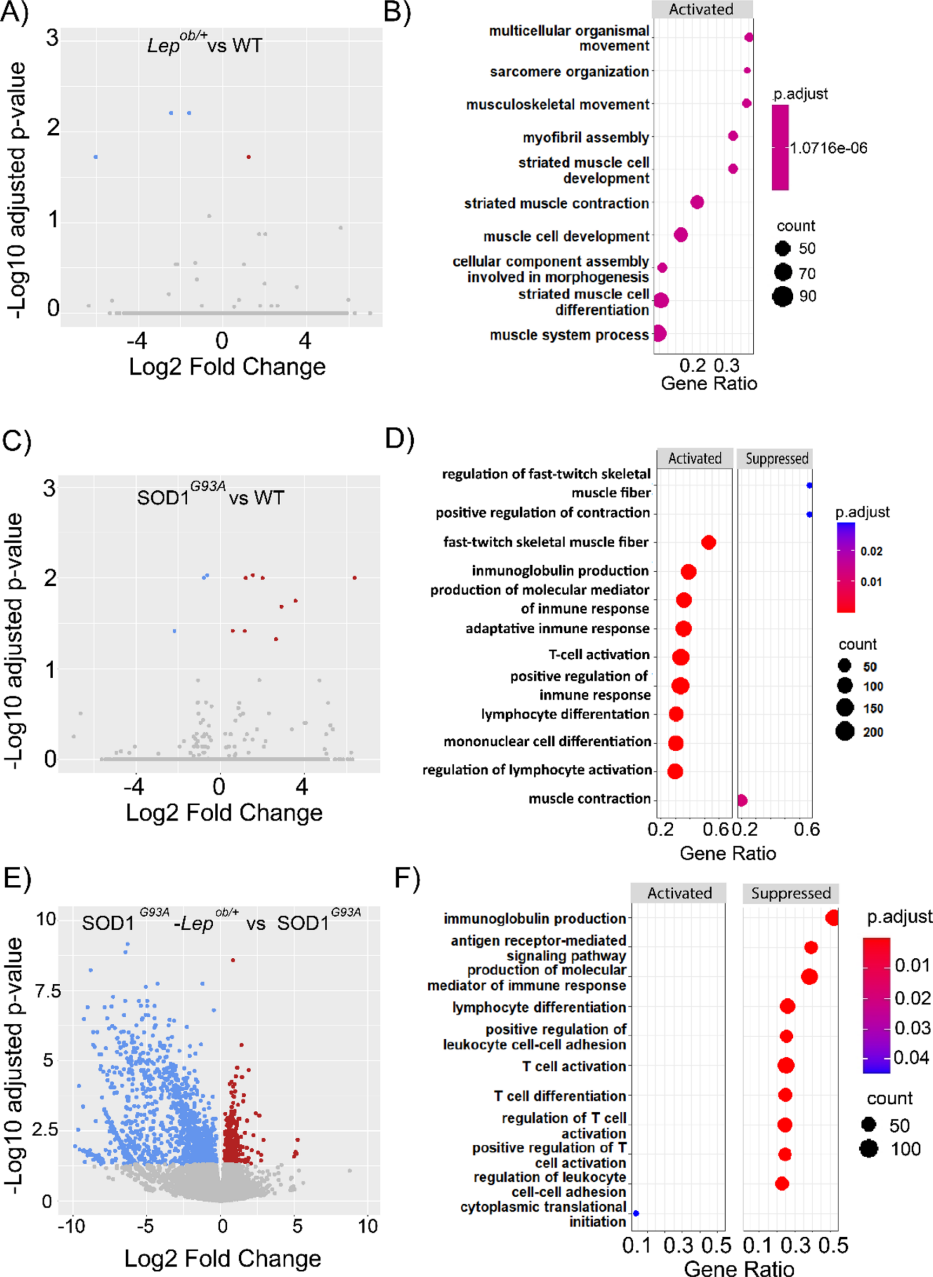
Transcriptomic profile in the iWAT of SOD1^G93A^ and leptin deficient mice. Volcano plots showing the DEGs identified by FDR < 0.05 in the iWAT of 90 days old female mice, comparing; (**A**) *Lep*^*ob/*+^ vs WT, (**C**) SOD1^G93A^ vs WT, and (**E**). SOD1^G93A^-*Lep*^*ob/*+^ versus SOD1^G93A^. Dots in blue denote genes that were downregulated and in red genes that were upregulated. Gray dots denote genes that are not significantly changed with the threshold of FDR < 0.05. (**B**, **D**, **F**) Dot plots showing dysregulated pathways identified by GSEA with FDR < 0.05. The size of the dots was proportional to the number of the genes implicated in the pathway and the color of the dots represented the significance related to the value of the FDR.

First, we run a transcriptomic analysis of the iWAT of the four groups of interest female mice (n = 4) at early symptomatic disease stages (P90) in order to identified differentially expressed genes (DEGs) with a false discovery rate (FDR) < 0.05. The transcriptional changes that the leptin haploinsufficiency induced directly on the iWAT tissue, by comparing the iWAT transcriptome of *Lep*^*ob/*+^ mice to the WT mice, showed no significant alterations (Fig. 3A), even though the *Lep*^*ob/*+^ mice, and marginally their iWAT, weighed more than their wild-type littermates (Fig. 2C,D). Only five DEGs were identified comparing the iWAT of the *Lep*^*ob/*+^ to WT mice. Of those, four were downregulated (*Aqp5*, *Itagv*, *Gm128*, *Gm4613*) and one upregulated (*Ddit4*). The GSEA pathway analysis showed a few activated processes related to multicellular organization and organization including muscle differentiation (Fig. 3B).

Next, we evaluated the transcriptional alterations in the iWAT of SOD1^G93A^ mice, compared to WT, and identified 14 DEGs (11 up and 3 down) with threshold FDR 0.05 (Fig. 3C) and 710 DEGs with p value < 0.05 (Supplementary Fig. 1A). In order to identify the pathways altered by SOD1 mutation in the iWAT, we run pathway analysis by ORA and GSEA. The analysis of biological processes enriched by the ORA technique with DEGs showed that 9 of the top 10 deregulated processes are related to RNA splicing (Supplementary Fig. 1B). Furthermore, the pathway analysis performed by the GSEA technique, showed an upregulation of several processes related to the immune system: "immunoglobulin production”, “lymphocyte differentiation”, "production of molecular mediator of immune response", “T-cell activation” (Fig. 3D). These data suggest a mild activation of the immune response in the iWAT of SOD1^G93A^ mice even at early disease stages, which could partly explain why these mice had more iWAT than their wild-type littermates, despite weighing less.

Lastly, we evaluated the transcriptional effect of lowering the levels of leptin in the iWAT of SOD1^G93A^ mice. The comparison between the transcriptome of SOD1^G93A^ and SOD1^G93A^–*Lep*^*ob/*+^ mice identified 1793 DEGs with FDR < 0.05, with a clear predominance of inhibited genes (398 up and 1394 down) (Fig. 3E) and 5000 DEGs with p value < 0.05 (Supplementary Fig. 1C). The ORA analysis showed that the top 10 dysregulated pathways are processes related to the immune system "T-cell activation", "adaptive immune response", and "lymphocyte differentiation" (Supplementary Fig. 1D). The GSEA analysis showed these same processes to be the most dysregulated and determined that they were inhibited (Fig. 3F). Up to 483 of the 1793 identified DEGs in the iWAT of SOD1^G93A^–*Lep*^*ob/*+^ mice were associated with the GO term immune system process (GO:0002376), and up to 204 of them were involved in lymphocyte activation (GO:0046649). Most of these genes were strongly inhibited, with up to 61 genes having a log2 fold change < − 5.

### Leptin deficiency countered the lymphocyte activation processes in the iWAT of SOD1^G93A^ mice

**Figure 4:**
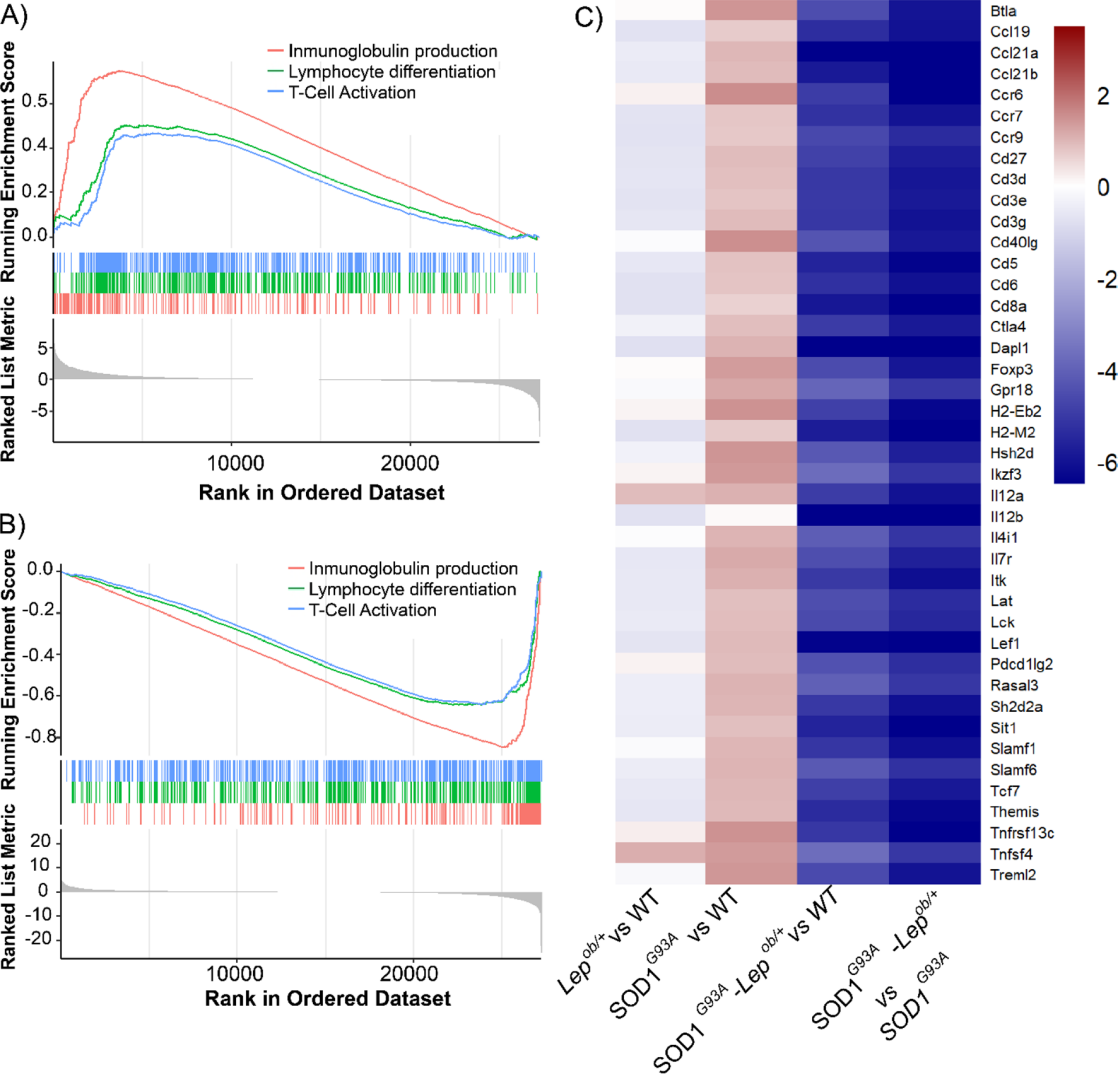
Analysis of alterations in the expression of immune pathways in the iWAT (**A**) Enrichment score plot of the GSEA results for “Immunoglobulin production”, “Lymphocyte differentiation”, “T-cell activation” in the iWAT SOD1^G93A^. (**B**) Enrichment score plot of the GSEA results for “Immunoglobulin production”, “Lymphocyte differentiation”, “T-cell activation” in SOD1^G93A^–*Lep*^*ob/*+^ iWAT (**C).** Hierarchical clustering heatmap for top deregulated genes involved in T cell activation in the iWAT. Columns represent the comparison between the different groups of interest (*Lep*^*ob/*+^vs WT; SOD1^G93A^ vs WT and SOD1^G93A^–*Lep*^*ob/*+^ vs SOD1^G93A^), and rows represent each gene found differentially expressed related to the lymphocyte T activation process. Red color represents overexpression and dark blue downregulation of genes related to lymphocyte T activation processes in the iWAT.

We next looked deeper into the specific transcriptional activation of the immune response caused by the SOD1^G93A^ transgene and whether those might be countered by the leptin deficiency, iWAT of SOD1^G93A^ mice. We selected commons immune processes deregulated in SOD1^G93A^ mice and SOD1^G93A^–*Lep*^*ob/*+^ in the iWAT (“immunoglobulin production”, “lymphocyte differentiation” and “T-cell activation”) and plotted the distribution of the fold change of the genes involved in these pathways (Fig. 4A). The enrichment score plot indicates that most of these pathways were activated in the iWAT of SOD1^G93A^ mice, and the opposite inhibition was observed when reducing the levels of leptin in the context of SOD1^G93A^ (SOD1^G93A^–*Lep*^*ob/*+^) (Fig. 4B).

Among all the immune system processes found, the “T-cell activation” process was the most notable. Thus, we identified the highly altered genes involved on these processes and looked for their profile distribution, represented in a hierarchical clustering (Fig. 4C). The analysis evidenced that the effect of the two mutations combined had a stronger effect on those genes analyzed than any of the mutations alone (SOD1^G93A^ vs WT, or *Lep*^*ob/*+^ vs WT). Leptin deficiency alone (*Lep*^*ob/*+^ vs WT) had a mild but evident inhibition of the genes involved in the T cell activation pathway in the iWAT. The SOD1^G93A^ transgene induced an upregulation (shown in red colour) of several of those genes. The combination of the two mutations had a stronger inhibition of the genes related to lymphocyte T activation pathway in the iWAT (SOD1^G93A^ vs SOD1^G93A^–*Lep*^*ob/*+^). Among the genes identified were chemokines (*Ccl19*, *Ccl21a*, *Ccl21b*, *Ccr6*, *Ccr7*, *Ccr9*), surface antigens (*Cd3d*, *Cd3e*, *Cd3g*, *Cd5*, *Cd6*, *Cd8a*, *Cd40lg*), histocompatibility antigen (*H2-Eb2*, *H2-M2*), interleukins (*Il12a*, *Il12b*, *Il4i1b*, *Il7r*), immunoglobulins (*Btla*, *Ctla4*, *Ighd*, *Ighg1*, *Igkj5*) and tumor necrosis factor (*Tnfrsf13C*, *Tnfrsf4*).

### The transcriptional effect of SOD1^G93A^ mutation is higher in the spinal cord than in the iWAT

Since the spinal cord is the tissue primarily affected by the SOD1^G93A^ mutation, we evaluated if the systemic effect of lowering leptin levels would have a direct impact on the spinal cord. Thus, in parallel, we run a transcriptomic analysis of the spinal cord on the same mice as in the transcriptomic analysis of the iWAT. The expression of the leptin receptor in the spinal cord is considerably low as reported in Protein Atlas database (https://www.proteinatlas.org/ENSG00000116678-LEPR/tissue) and some works^24,26^. We confirmed that the expression of leptin was much higher (nearly 19 times more) in the iWAT than in the spinal cord (which expression is basically null) (Supplementary Fig. 2A). On the other hand, the differences of the gene expression of the leptin receptor in those two tissues (Supplementary Fig. 2B) were not that obvious. In addition, we verified the expression of the protein of the leptin receptor in the spinal cord. The leptin receptor protein was present in all spinal cords from all genotypes, but the mice with leptin haploinsufficiency (*Lep*^+*/−*^*)* showed slight upregulation of the receptor expression, which might suggest a compensatory mechanism that is not present in the SOD1^G93A^ *Lep*^+*/−*^ mice (Supplementary Fig. 2C,D).

**Figure 5:**
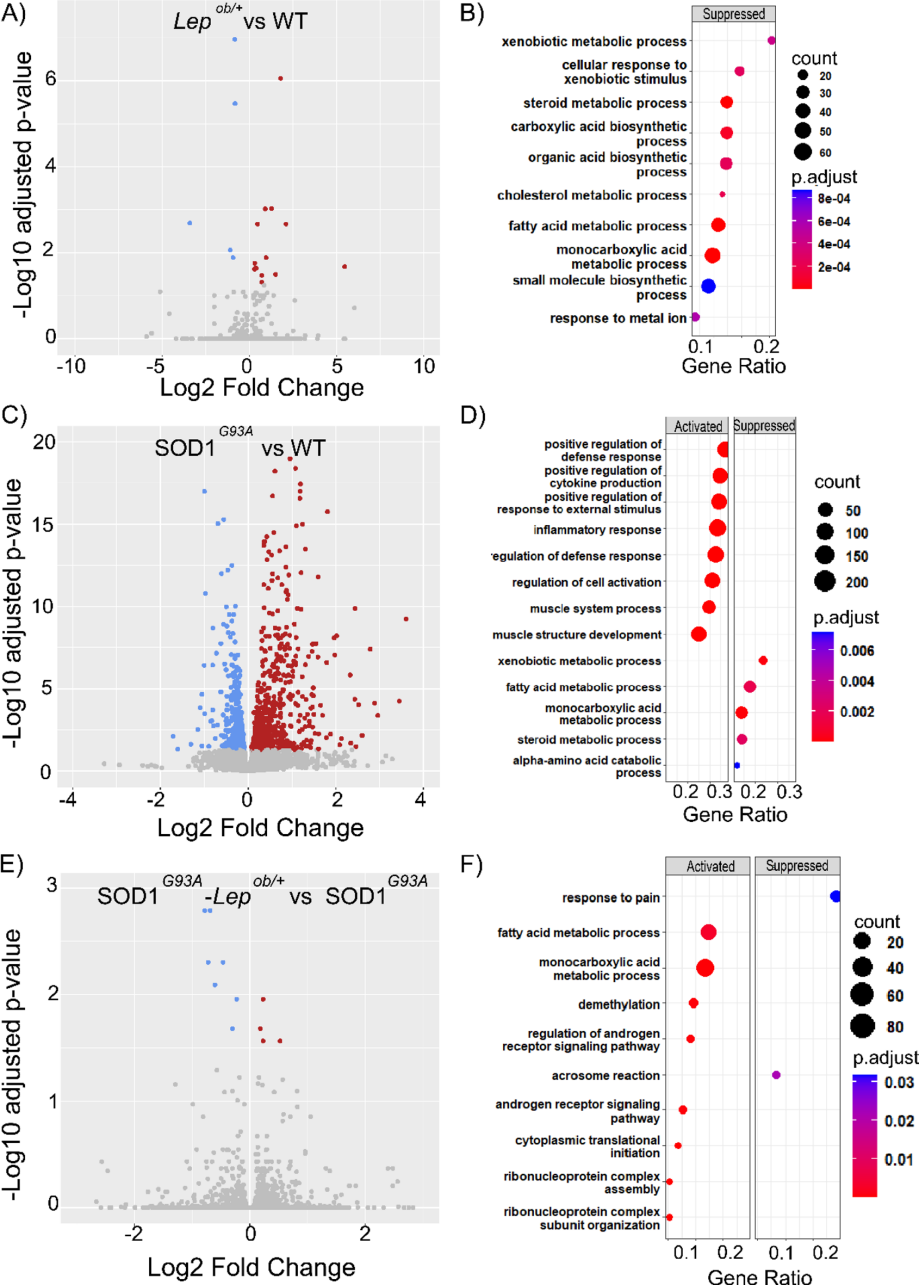
Transcriptomic profile in the SPC of SOD1^G93A^ and leptin deficient mice. Volcano plots showing the DEGs identified by FDR < 0.05 in the SPC of 90 days old female mice, comparing; (**A**) *Lep*^*ob/*+^vs WT, (**C**) SOD1^G93A^ vs WT, and (**E**). SOD1^G93A^–*Lep*^*ob/*+^ versus SOD1^G93A^. Dots in blue denote genes that are downregulated and in red genes that are upregulated. Gray dots denote genes that are not significantly changed with the threshold of FDR < 0.05. (**B**, **D**, **F**) Dot plots showing deregulated pathways identified by GSEA with FDR < 0.05. The size of the dots are proportional to the number of the genes implicated in the pathway and the color of the dots represent the significance related to the value of the FDR.

As with the iWAT, we first evaluated the effect of the leptin deficiency alone in the transcriptome of the lumbar spinal cord (SPC) (*Lep*^*ob/*+^ vs WT) and found very mild effects, with 21 DEGs identified (16 up and 5 down) at FDR < 0.05 (Fig. 5A). The GSEA pathway analysis showed a down regulation of processes related to metabolism, such as “fatty acid metabolic process", “monocarboxylic acid metabolic process” and “cholesterol metabolic process” (Fig. 5B). The ORA analysis revealed other altered processes, mainly in the PI3K signaling pathway. Interestingly, the "negative regulation of cytokine production" pathway was among the top 10 GO terms altered, which is consistent with the anti-inflammatory effect of leptin deficiency (Supplementary Fig. 3A).

The transgene SOD1^G93A^ had a very strong effect on the SPC gene expression profile, as we previously shown (from Fernández-Beltrán LC et al. 2021). We identified 1174 DEGs (639 up and 535 down) (Fig. 5C, adapted from Fernández-Beltrán LC et al. 2021). Similar to the iWAT transcriptome of SOD1^G93A^ mice, the SOD1^G93A^ transgene induced more upregulation of genes than inhibition. In the GSEA analysis we also found, among the top dysregulated biological pathways, several pathways related to an activation of immune system, such as: “positive regulation of defense response”, “positive regulation of cytokine production”, “inflammatory response” (Fig. 5D). On other hand, top downregulated processes were related to metabolic processes such as: fatty acid, steroids, and alpha amino acids. Similar results were found with the ORA analysis in relation to immune system activation: leukocyte activation, tumor necrosis factor, and phagocytosis (Supplementary Fig. 3B).

In the spinal cord of the SOD1^G93A^ the effect of lowering leptin levels was very mild. Compared to the SOD1^G93A^ alone, the transcriptome of the SPC of SOD1^G93A^–*Lep*^*ob/*+^ identified 18 DEGs with FDR > 0.05 (9 up and 9 down) (Fig. 5E). These results were expected since the effect of lowering the leptin levels might be mostly indirect on the spinal cord, and from systemic metabolic changes. Looking at the most relevant biological processes and pathways altered by the combination of the two mutations (SOD1^G93A^–*Lep*^*ob/*+^)*,* the GSEA analysis identified an activation of transcription-translation processes (“Demethylation”, “ribonucleoprotein complex assembly” and “Cytoplasmic translational initiation”) and metabolic processes (“Fatty acid metabolic process” and “Monocarboxylic acid metabolism process”) (Fig. 5F). These metabolic processes were downregulated in both *Lep*^*ob/*+^ and SOD1^G93A^ mice when compared with WT.

### Leptin deficiency had a mild effect on maintaining the regulation of fatty acid metabolism and no effect on inflamation in the spinal cord of SOD1^G93A^ mice

**Figure 6:**
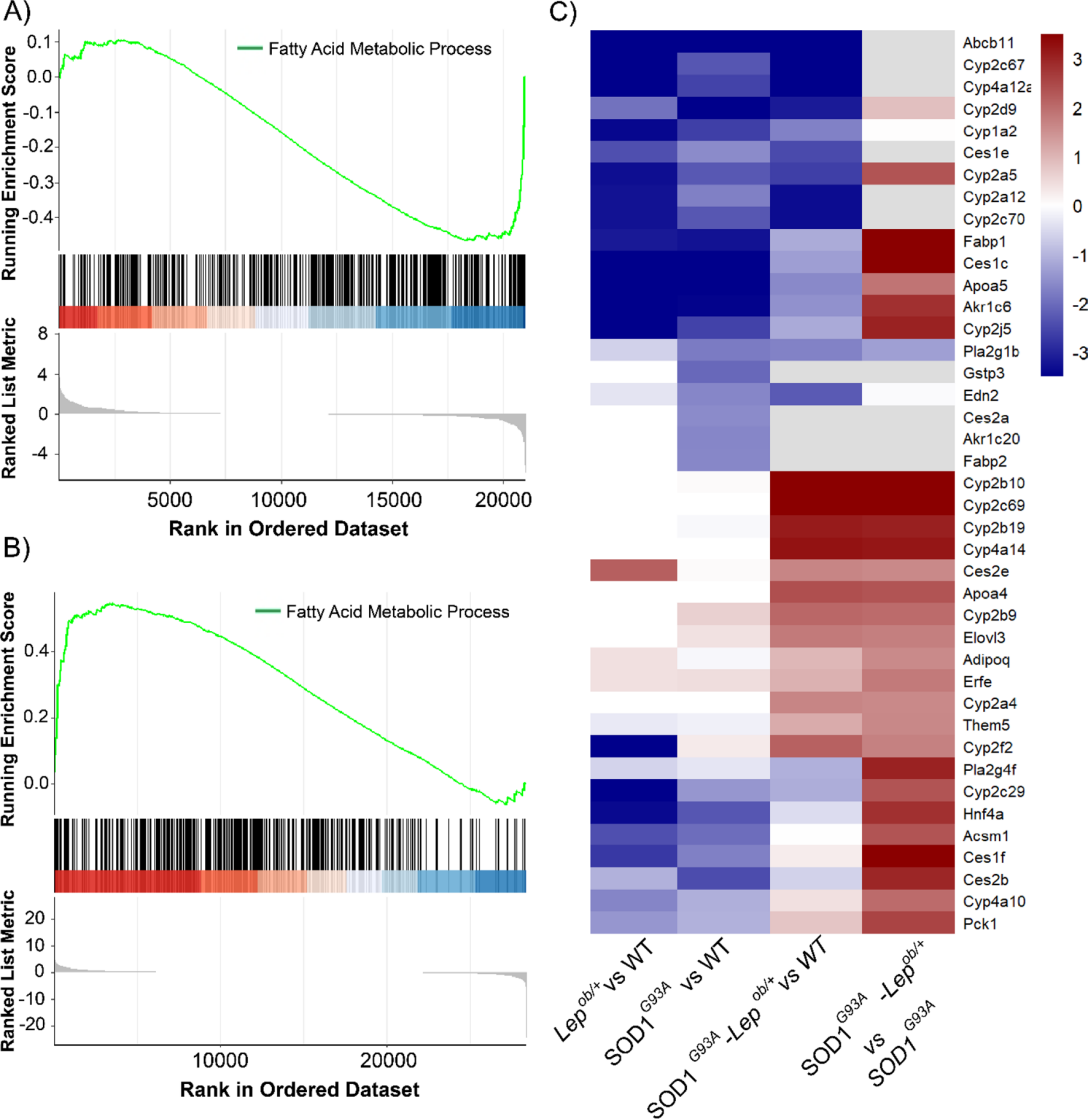
Analysis of alterations in the expression of fatty acid metabolism in the SPC (**A**). Enrichment score plot of the GSEA results for “Fatty acid metabolic process” in the SPC SOD1^G93A^. (**B**) Enrichment score plot of the GSEA results for “Fatty acid metabolic process” in the SPC in SOD1^G93A^–*Lep*^*ob/*+^ iWAT. (**C**) Hierarchical clustering heatmap for top deregulated genes involved in fatty acid metabolism in SPC. Columns represent the comparison between the different groups of interest (*Lep*^*ob/*+^vs WT; SOD1^G93A^ vs WT, and SOD1^G93A^–*Lep*^*ob/*+^ vs SOD1^G93A^), and rows represent each gene found differentially expressed related to the fatty acid metabolic process. Red color represents overexpression and dark blue downregulation of genes related to fatty acid metabolic process processes in the SPC.

Lowering the levels of leptin had minimum effect on the transcriptional inflammatory response in the SPC in the SOD1^G93A^ background (comparing SOD1^G93A^–*Lep*^*ob/*+^ vs SOD1^G93A^) (Supplementary Fig. 4). Interestingly, other metabolic pathways seemed to be changed by the approach. The pathway analyses in the SPC of the different groups also revealed that metabolism processes were dysregulated not only by SOD1^G93A^ but also when combined with leptin deficiency (Fig. 5B,D,F). The “fatty acid metabolism” (GO: 0006631) was the common metabolic process dysregulated in SOD1^G93A^ mice and in SOD1^G93A^–*Lep*^*ob*/+^ in the SPC. We identify the genes altered on this process and plotted them in a distribution based on their up or down regulation by fold change (FC). The genes involved in fatty acid metabolism tend to be inhibited in the SPC of SOD1^G93A^ mice (Fig. 6A), while leptin deficiency when placed in SOD1^G93A^ background, showed increased expression of these same fatty acid genes (Fig. 6B). Finally, we performed a hierarchical clustering analysis on those genes involved in fatty acid metabolism comparing the expression profile among the four different groups. Leptin deficiency in the spinal cord of SOD1^G93A^ mice corrected the inhibition of some of the genes involved in fatty acid metabolism (Fig. 6C).

These results support the concept that lowering systemic leptin levels affected the transcriptional profile of the spinal cord, although milder than in the iWAT, and through different pathways. These differences between the tissues could be reflecting the fact that the effect of leptin haploinsufficiency might be direct in the iWAT and seemed indirectly in the SPC.

## Discussion

ALS patients typically have normal or low body mass index (BMI), and lose weight as the disease progresses, classically associated with a loss of lean mass but also fat mass, especially in the final stages of the disease^4^. In order to slow or preserve the weight loss and disease progression, several attempts have been made with some modest results, including lowering the levels of the anorexigenic hormone leptin in the SOD1^G93A^ mice. Interestingly, the positive effects of genetically reducing the levels of leptin in SOD1^G93A^ mice was sexually dimorphic, showing some beneficial effect in females (i.e., extending survival) but not in male SOD1^G93A^ mice^24^. The molecular mechanism by which leptin deficiency was exerting the beneficial effect by preserving the body weight was not known, and whether the effect was directly on the spinal cord or indirect through general metabolic changes.

We first clarified the previous controversial results about the levels of leptin in ALS patients. We found that the circulating levels of leptin in women and men suffering from ALS are different. Since leptin is mainly produced in the adipose tissue, the amount of circulating leptin is normally associated with the amount of body fat under normal conditions. Thus, since ALS patients are thought to lose fat mass as disease progresses, the levels of leptin in ALS patients are expected to follow proportionally to the amount of fat. Indeed, that is what was found in men with ALS, but in the case of the women, the same trend was not true. This sexual dimorphism is supported by other larger studies, where they found lower levels of leptin in men and no changes in women with ALS^22^. Interestingly, in non-pathological situations, leptin levels in women can double the men´s levels^27,28^. The first explanation proposed was due to the distribution of fat. Women have a greater amount of subcutaneous fat that produces more leptin while men have more abdominal fat^29^. However, other studies suggest that the distribution of fat does not explain the dimorphism in leptin levels^30^ but rather the sexual hormones that would regulate the transcription of leptin^31^. This sexual dimorphism in the circulating level of leptin is also replicated in SOD1^G93A^ mice^24^ (Fig. 2B). Thus, we evidenced that any approach towards modifying leptin or the fat mass and weight in ALS patients and mice, should be carefully taken the sex into account, as the sexual metabolic dimorphism would give contradictory results. For example, since many ALS patients have low levels of leptin, especially men and all towards final stages of disease, other attempts towards increasing leptin have been tried^25^. This could be even more detrimental in women with ALS, whose leptin levels are normally more elevated, but also in men, since leptin administration could induce the acceleration of the metabolisms (more hypermetabolism) and satiety (reducing food consumption), which could result in an accelerated weight loss.

It is important to note that very few studies, mainly those conducted in mice, correlated the levels of circulating leptin with the amount of adipose tissue as well as stratified by sex. In our present study, we also added that further stratification, considering the amount of adipose tissue in the different sexes. Altogether, our data and the others, suggest that the fat tissue seems also altered in ALS, and differentially in men and women, and that is then reflected in the levels of circulating leptin. In the present study, we followed the same strategy of stratifying by sex, to corroborate that sex is one of the main determinants in the general metabolism of the body with huge influence in the disease. Again, the ratios of men and women who suffer ALS are not equal (1, 5:1), and the differences in body metabolism between the two sexes could be one of the explanations.

One of the main roles of leptin is the regulation of the systemic energy metabolism through the hypothalamus. In this context, the hypometabolic effect induced by lowering leptin levels was considered as a potential strategy to treat ALS. Indeed, the SOD1^G93A^–*Lep*^*ob/*+^ mice had reduced energy expenditure, with less motor deficit and extended survival, although these effects were only found in females^24^. However, to date, the effects of those systemic metabolic changes driven by the hypothalamus by leptin deficiency, and the molecular pathways induced by these systemic changes in the spinal cord and other tissues of female SOD1^G93A^ mice, remains unknown.

Leptin is secreted by the adipose tissue and has been extensively studied in obesity, where leptin can be highly produced and negatively contribute to the inflammation of the adipose tissue, further impairing the normal function of the adipose tissue in obese patients. According to this, we found that there were inflammatory processes activated in the iWAT of the female SOD1^G93A^ mice, which are the ones with higher amounts of circulating leptin in the body, and bigger fat depots. Interestingly, the effect of leptin haploinsufficiency in the iWAT and in the context of the SOD1^G93A^ mutation was the inhibition of those inflammatory processes. The regulation that leptin exerts on the immune system is because most immune cells express the leptin receptor. When its ligand binds, the JAK proteins (Janus Kinases) become autophosphorylated and in turn phosphorylate the STAT proteins (signal transducers and activators of transcription). Other signaling cascades such as MAPK (mitogen-activated protein kinase) and PI3K (phosphatidylinositol 3-kinase) are also activated by leptin signaling. The activation of these signaling cascades has numerous effects on the cells of the immune system: it increases the proliferation of circulating monocytes^32^, promotes cell survival of eosinophils and basophils^33^, increases cytotoxicity of NK cells^34^ and the production of proinflammatory cytokines from T lymphocytes^35^. Consistently with our results, in the iWAT of SOD1^G93A^–*Lep*^*ob/*+^ mice we observed a significant downregulation of cytokines, interleukins and their receptors, and tumor necrosis factors in adipose tissue.

It is worth mentioning the little anti-inflammatory effect of leptin haploinsufficiency in the context of the SOD1^G93A^ mutation in the spinal cord of the mice. Among many other reasons, it could be because there are very few leptin receptors in the spinal cord, and the type of receptors could be different to the ones in the iWAT. In addition, the strong degenerative process induced by the SOD1^G93A^ mutation in the spinal cord causes such an inflammation state that might be too strong to be reverted by leptin haploinsufficiency. Still, it seems that there is another effect of leptin haploinsufficiency in the context of the SOD1^G93A^ mutation in the spinal cord, which is the regulation of fatty acid metabolism, and that could be an indirect effect of the changes in the systemic body metabolism. Thus, we believe that the relevance of this work is that it remarks the importance of the whole-body system in these complex disorders, and how we need to take care of other tissues and the systemic metabolism in order to have a better disease prognosis. Further studies are needed to disentangle the link, or multiple links, between the effect of the general body metabolism and the spinal cord.

There are limitations in the work. One is the low number of patients in the study, although the data clearly corroborate previous large epidemiological studies, with similar results. Another limitation is that all these differences are measured in one early –disease time point in the mice, and in that way, we cannot exclude that all those results could be changing along the disease progression.

In conclusion, the anti-inflammatory effects of decreasing leptin levels demonstrated in this work could be an interesting therapeutic target for some ALS patients. Here we bring up the importance of considering the sex in every metabolic study. In particular, the sexual dimorphisms found in relation to the levels of circulating leptin and fat tissue, which could be used to stratify and might be critical for the inclusion of ALS patients in different clinical trials. These considerations should be included in future personalized treatments.

## Methods

### Participants

Thirty-five participants were recruited between October 2021 and April 2023. Among them, sixteen were healthy participants (9 men and 7 women), and nineteen were patients (9 men and 10 women) diagnosed with ALS in the Neurology Department of Hospital Clínico San Carlos (Madrid, Spain). All the patients met the revised El Escorial diagnostic criteria of clinically definite, probable ALS. All the participants were excluded of severe metabolic disease. Additional demographic information of the participants can be found in Supplementary table 1. The estimation of the amount of subcutaneous adipose tissue was done using the skinfold thickness. Skinfold thickness was measured in triplicate on the right side of the body using a Holtain® skinfold caliper at the following sites: triceps, subscapular crest and iliac crest. The sum of the mean thickness of each skin fold was used in the analyses. The present study was approved by the ethics committee from Hospital Clínico San Carlos (reference: 19/524-E) in accordance with EU guidelines and regulations. Participants provided written informed consent prior to research participation.

### Animals

The SOD1^G93A^ mouse strain [B6.Cg-Tg(SOD1-^G93A^)1Gur/J)] carries a high copy number (approximately 25 copies) of the SOD1 transgene, were obtained from Jackson Laboratories (Bar Harbor, Maine, USA) and were maintained on a C57BL/6J background (purchased from Charles River) in our lab. We also obtained mice heterozygous for the obese gene from (B6.Cg-Lepob/J, Jackson Laboratories) and were also maintained on a C57BL/6J background. SOD1^G93A^ mice were genotyped using conventional PCR, as well as controlling for the transgene copy number, using quantitative PCR (IMR0113 and IMR0114 designed by Jackson labs). *Lep*^*ob/*+^ mice were genotyped by quantitative PCR to identify mutant and WT alleles with probes (Primers: Fw: GCAGTCTATCAACAGGTCCTCA and Rv: TTGGAGAAGGCCAGCAGA. Probes: Lep-OB_WT_FAM GAATCTCCGAGACCTCCT, 5'FAM-'3BHQ1 and Lep-Mutant-JOE AATCTCTGAGACCTCCT, 5′JOE-3′BHQ1). Mice were kept on auto-ventilated cages with food *ad libitum* on a 12 h light–dark cycle. Mice were weighed weekly and humanely sacrificed when they reached paralysis of hind limbs or 20% of weight loss. All animal procedures were approved by the ethical committee of animal care and use of the Hospital Clínico San Carlos and in accordance with the European and Spanish regulation (2010/63/EU and RD 1201/2005).

### Leptin measurement

Participants were at least 4 hours fasted prior blood collection. Blood samples were collected in SSTII serum separator gel tube (Ref. 367953). Blood was allowed to coagulate for 20 mins at 4 °C and spun at 3000×*g* for another 10 min. Serum was collected and stored in − 80 degrees until use. Leptin concentrations were evaluated in duplicate using commercially available enzyme-linked immunosorbent assay kit (Quantikine ELISA Human Leptin, R&D Systems), in accordance with the manufacturer’s instructions.

Mice retro-orbital bleeds were performed after 4 h fasting under terminal anesthesia at 90 days of age (n = 5), and the blood was collected in lithium-heparin tubes, and spun at 4000×*g* for 8 min at 4 °C to obtain plasma. Leptin concentrations were evaluated in duplicate using commercially available enzyme-linked immunosorbent assay kit (Rat/Mouse Leptin ELISA kit Cat. #EZML-82K, Millipore, Missouri, USA), in accordance with the manufacturer’s instructions.

### RNA extraction and sequencing

RNA was isolated from dissected iWAT (n = 4) and lumbar region of spinal cords (n = 5) of wild-type, *Lep*^*ob/*+^, SOD1^G93A^ and SOD1^G93A^–*Lep*^*ob/*+^ female mice at 90 days, using Qiazol followed by the mini lipid tissue RNAeasy kit (Qiagen, Hilden, Germany). The RNA used for sequencing had a RIN value above 8–9 in the Bioanalyzer. The samples were sent to the company NIM Genetics (Madrid, Spain) for sequencing. The quality control of the samples was achieved with TapeStation (Agilent Technologies, Santa Clara, CA, USA) followed by quantification using the fluorometric system Qubit (Thermo Fisher Scientific, Waltham, MA, USA) cDNA libraries were made using TruSeq Stranded mRNA Library Prep and sequenced on NovaSeq 6000 (all Ilumina, Inc., San Diego, CA, USA) producing paired-end 100 bp reads.

### RNA-seq data processing

Quality control of FastaQ files was performed using FastQC (https://www.bioinformatics.babraham.ac.uk/projects). Low-quality reads (Phred quality score < 30) and reads too short (length < 30 pb) were removed using Fastp^36^. The alignment to the genome (10 mm mouse reference genome) was achieved using HISAT2^37^. The expression quantification of genes was carried out using FeatureCounts^38^. Only uniquely mapped reads were used for the analysis of differential gene expression quantification with DESeq2^39^. Raw p-values were adjusted by the Benjamini–Hochberg false discovery rate (FDR) method and the adjusted p-values less than 0.05 were considered statistically significant.

Volcano-plots, PCA analysis, and heatmaps were generated using R and the following packages: “DESeq2”^39^ and “pheatmap” (https://CRAN.R-project.org/package=pheatmap).

### Over representation analysis (ORA)

ORA analysis compares a set of interesting genes or proteins (test set) to a background distribution (reference set) concerning a certain biological category (e.g. a metabolic pathway). The distribution of test set genes that were contained in the considered biological category were compared to the genes of the reference set having this property. If more genes in the test set belonged to the considered biological category than expected, this category was enriched or over-represented, otherwise the category was depleted or under-represented in the test set. The ORA was performed using the clusterProfiler package (v3.16.1) in R. As input, it receives all (both up and down regulated) DEGs from DESeq2, obtained using the cut-off criteria for statistical significance p-adjusted value < 0.05.

### Gene set enrichment analysis (GSEA)

Gene set enrichment analysis (GSEA) is a genome-wide expression profile chip data analysis method for identifying functional enrichment through a comparison of genes and predefined gene sets (39). The GSEA was performed using the clusterProfiler package (v3.16.1) in R. As input, it receives all ranked genes by fold-change from DESeq2 analysis. The significance of enriched gene sets was estimated by false discovery rate (FDR) q-value < 0.05.

### Statistical analysis

Statistical analysis was performed using GraphPad Prism version 8.0.1. Two groups were compared with a single time point using Student's t test. Body weights between genotypes, at multiple time points, were compared using repeated measures/mixed models two-way ANOVA and multiple testing Bonferroni correction. Two groups were compared at multiple time points using 2-way ANOVA with Šídák's multiple comparisons post hoc test. Three or more groups were compared at a single time point using ordinary one-way ANOVA with Dunnett's post hoc test. See figure legends for sample size -n numbers; n numbers refer to biological samples (i.e., number of animals used in animal experiments). Statistical details of each experiment can be found in the figure legends. Significance is indicated with the following points: *p ≤ 0.05, **p ≤ 0.01, ***p ≤ 0.001, ****p ≤ 0.0001. BioRender.com was used to create original diagrams/figures.

### Supplementary Information

Supplementary Information.

## Data Availability

Raw and processed RNA-sequencing data in this study were deposited in the NCBI Gene Expression Omnibus (GEO, http://www.ncbi.nlm.nih.gov/geo) with the following accession number: GSE248515 (for the iWAT data), and GSE184484 (for the spinal cord).
