# Supplementary material for: Leptin haploinsufficiency exerts sex-dependent partial protection in SOD1G93A mice by reducing inflammatory pathways in the adipose tissue
Source: Sci Rep. 2024 Feb 1;14:2671. doi: 10.1038/s41598-024-52439-z (PMC10834470; doi:10.1038/s41598-024-52439-z)
Supplement: Supplementary file 1 — Supplementary Information. [file 41598_2024_52439_MOESM1_ESM.docx]

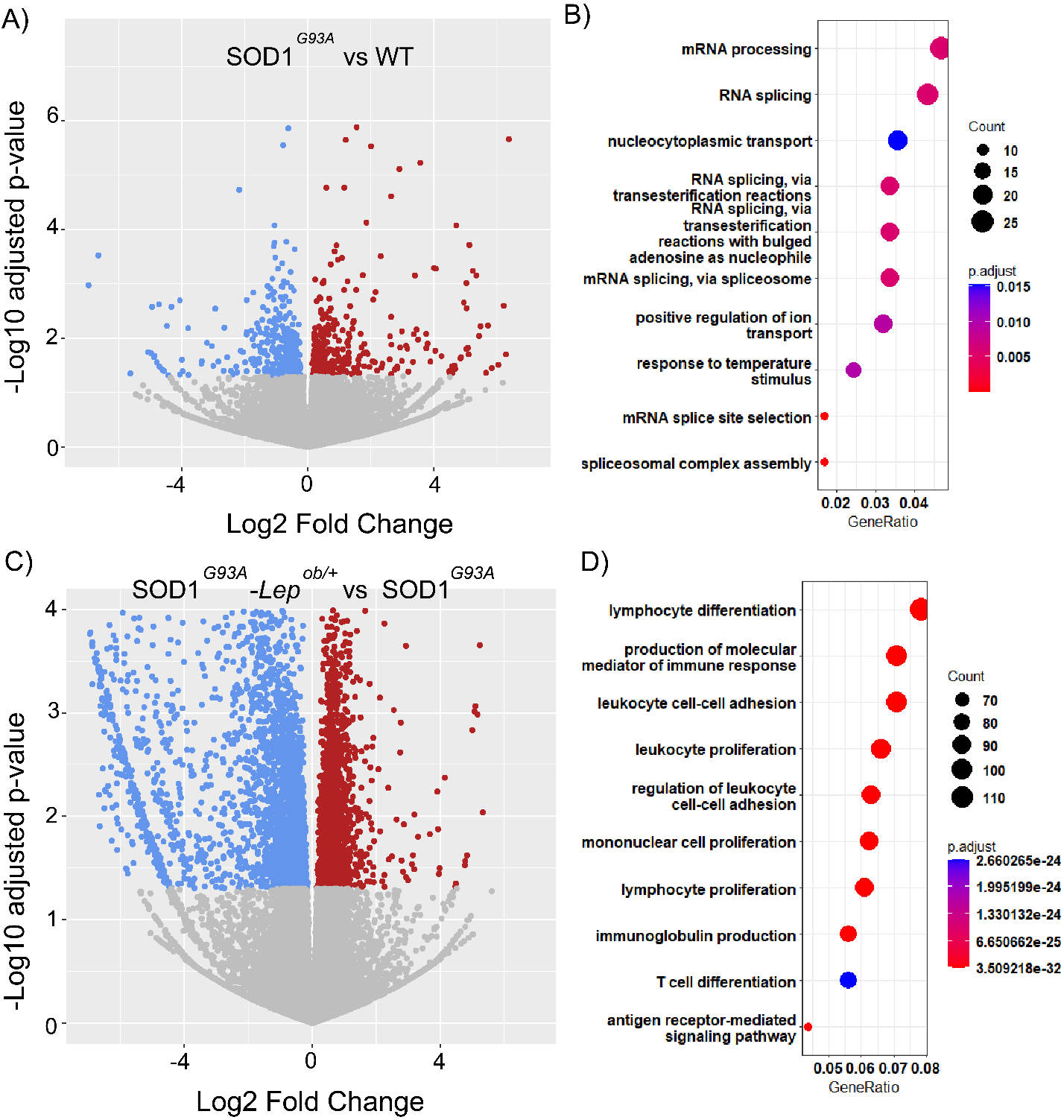


**Supplementary Figure 1**. Transcriptomic profile in the iWAT of SOD1^G93A^ and leptin deficient mice. Volcano plots showing the DEGs identified by p value < 0.05 in the iWAT of 90 days old female mice, comparing; **A.** SOD1^G93A^ vs WT, **and C.** SOD1^G93A^-Lep^ob/+^ versus SOD1^G93A^. Dots in blue denote genes that are downregulated and in red genes that are upregulated. Gray dots denote genes that are not significantly changed with the threshold of p value < 0.05. **B,D.** Dot plots showing deregulated pathways identified by ORA using DEGs with p value <0.05. The size of the dots was proportional to the number of the genes implicated in the pathway and the color of the dots represented the significance related to the value of the FDR.


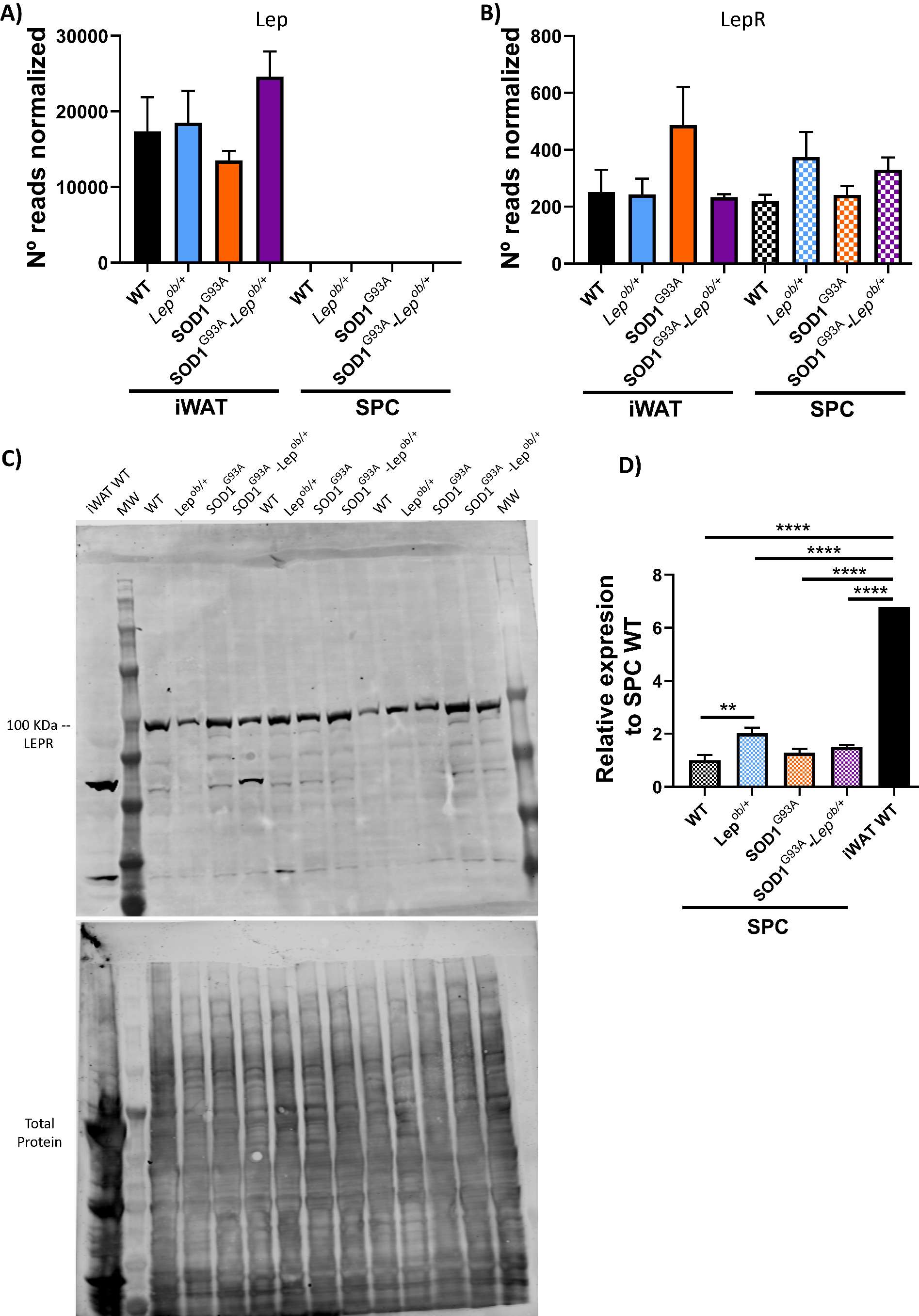


**Supplementary Figure 2**. Analysis of leptin (*Lep*) and leptin receptor (*LepR*) expression in iWAT and spinal cord of female mice. **A.** Normalized number of counts identified by the RNA-seq of *Lep* gene in iWAT and spinal cord.  **B.** Normalized number of counts identified by the RNA-seq of *LepR* gene in iWAT and spinal cord. (n= 5 per genotype group). **C, D.** Western blot of Leptin receptor protein expression in the spinal cord of female p90 mice (n = 3 per genotype).


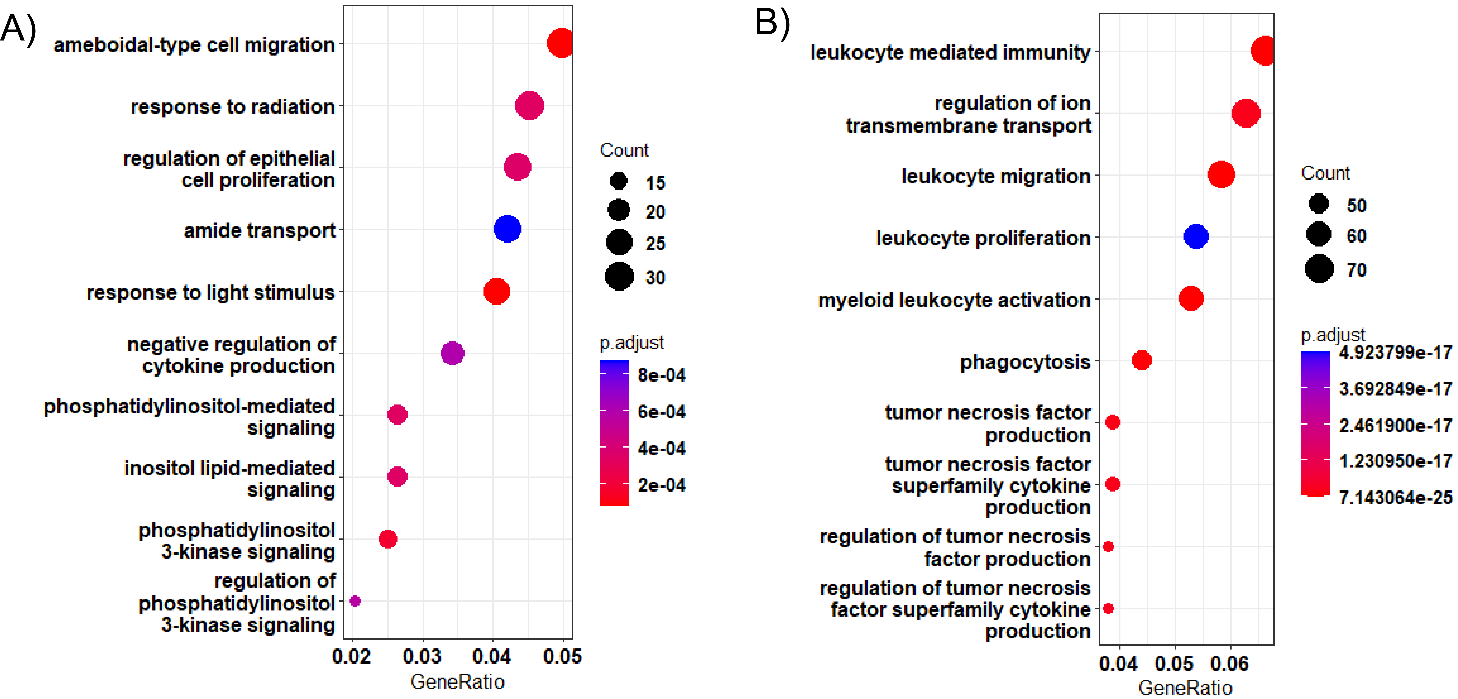


**Supplementary Figure 3**. Enriched biological pathways analysis in the SPC of SOD1^G93A^ and leptin deficient mice. **A and B**. Dot plots showing deregulated pathways identified by ORA using DEGs with p value <0.05. The size of the dots dwas proportional to the number of the genes implicated in the pathway and the color of the dots represented the significance related to the value of the FDR.


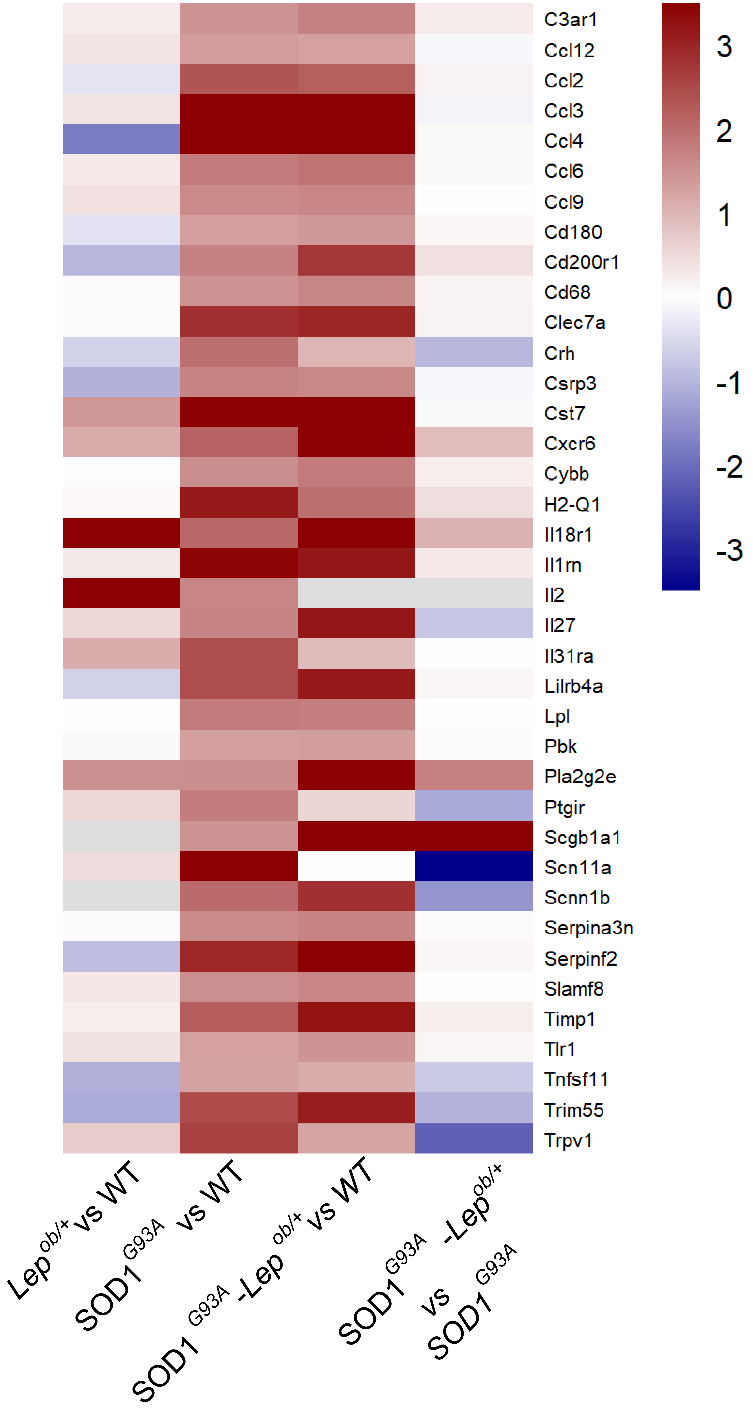


**Supplementary Figure 4.** Analysis of alterations in the expression of inmune pathwahys in the SPC. Hierarchical clustering heatmap for top deregulated genes involved in immune response in SPC. Columns represent the comparison between the different groups of interest (L*ep^ob/+^*vs WT; SOD1^G93A^ vs WT ; and SOD1^G93A^*-Lep^ob/+^* vs SOD1^G93A^), and rows represent each gene found differentially expressed related to the immune response. Red color represents overexpression and dark blue downregulation of genes related to immune response in the SPC.

**Supplementary Table 1.** Patient demographics data.

|  |  | **Control** | **ALS** |
| --- | --- | --- | --- |
| Total number (*n=35*) | | 16 | 19 |
| Male: Female | | 9: 7 | 9: 10 |
| Mean Age | | 61.2 | 60.5 |
| BMI (Male: Female) | <18.5 (underweight) | 1: 0 | - |
|  | 18.5- 25 (normal) | 2: 5 | 4: 4 |
|  | ≥25 (overweight) | - | 3: 0 |
|  | ≥30 (obese) | 3: 0 | - |
